# Feasibility of dynamic chest radiography to calculate lung volumes in adult people with cystic fibrosis: a pilot study

**DOI:** 10.1136/bmjresp-2022-001309

**Published:** 2023-05-05

**Authors:** Thomas Simon FitzMaurice, Caroline McCann, Dilip Nazareth, Scott Hawkes, Matthew Shaw, Paul Stephen McNamara, Martin Walshaw

**Affiliations:** 1 Department of Respiratory Medicine, Liverpool Heart and Chest Hospital NHS Foundation Trust, Liverpool, UK; 2 Institute of Life Course and Medical Sciences, University of Liverpool, Liverpool, UK; 3 Department of Radiology, Liverpool Heart and Chest Hospital NHS Foundation Trust, Liverpool, UK; 4 Institute of Infection and Global Health, University of Liverpool, Liverpool, UK; 5 Department of Pulmonary Physiology, Liverpool Heart and Chest Hospital NHS Foundation Trust, Liverpool, UK; 6 Research Department, Liverpool Heart and Chest Hospital NHS Foundation Trust, Liverpool, UK; 7 Department of Child Health (University of Liverpool), Institute in the Park, Alder Hey Children's Hospital NHS Foundation Trust, Liverpool, UK

**Keywords:** Cystic Fibrosis, Imaging/CT MRI etc, Respiratory Measurement, Lung Physiology

## Abstract

**Introduction:**

Dynamic chest radiography (DCR) is a novel, low-dose, real-time digital imaging system where software identifies moving thoracic structures and can automatically calculate lung areas. In an observational, prospective, non-controlled, single-centre pilot study, we compared it with whole-body plethysmography (WBP) in the measurement of lung volume subdivisions in people with cystic fibrosis (pwCF).

**Methods:**

Lung volume subdivisions were estimated by DCR using projected lung area (PLA) during deep inspiration, tidal breathing and full expiration, and compared with same-day WBP in 20 adult pwCF attending routine review. Linear regression models to predict lung volumes from PLA were developed.

**Results:**

Total lung area (PLA at maximum inspiration) correlated with total lung capacity (TLC) (r=0.78, p<0.001), functional residual lung area with functional residual capacity (FRC) (r=0.91, p<0.001), residual lung area with residual volume (RV) (r=0.82, p=0.001) and inspiratory lung area with inspiratory capacity (r=0.72, p=0.001). Despite the small sample size, accurate models were developed for predicting TLC, RV and FRC.

**Conclusion:**

DCR is a promising new technology that can be used to estimate lung volume subdivisions. Plausible correlations between plethysmographic lung volumes and DCR lung areas were identified. Further studies are needed to build on this exploratory work in both pwCF and individuals without CF.

**Trial registration number:**

ISRCTN64994816.

**WHAT IS ALREADY KNOWN ON THIS TOPIC**

- Dynamic chest radiography (DCR) is a quick, well-tolerated digital imaging technology capable of providing thoracic imaging alongside information on the movement of thoracic structures.

**WHAT THIS STUDY ADDS**

- We compared DCR lung areas with plethysmographic volumes in 20 adults with cystic fibrosis; DCR lung areas correlated well with corresponding plethysmographic lung volumes, and linear regression modelling could be used to calculate lung volume subdivisions.

**HOW THIS STUDY MIGHT AFFECT RESEARCH, PRACTICE OR POLICY**

- DCR might provide an alternative modality to calculate lung volume subdivisions in people with cystic fibrosis.

## Introduction

Lung volume subdivisions can provide important prognostic and diagnostic information about lung health in people with cystic fibrosis (pwCF). Inspiratory capacity (IC) is a subtle marker of lung function decline^1^ and exercise ability,^2^ total lung capacity (TLC) an important factor in donor/recipient matching and in understanding breathing patterns associated with transplantation,^3^ and functional residual capacity (FRC) may be a sensitive marker of early lung disease.^4^

However, the traditional method of lung volume measurement involves the assessment of flow over time, using forced manoeuvres which are not representative of real-world breathing, and involves the use of mouthpieces, face masks or enclosed chambers that alter normal ventilation.^5–8^ Whole-body plethysmography (WBP) can be problematic in claustrophobic individuals, spirometry requires complex repeated forced manoeuvres and both may be hazardous in those with transmissible respiratory infections.^9^ This has led to the exploration of other methods for measuring lung volumes, including plain chest radiographs,^10–12^ CT,^13–15^ MRI^14^ and chest wall kinematics.^16^ However, plain chest radiographs cannot measure lung volume subdivisions; although thoracic CT is capable of calculating accurate lung volumes,^17^ it confers an increased radiation dose^18 19^ and requires non-physiological supine positioning; MRI is time-consuming and difficult to acquire in claustrophobic or dyspnoeic individuals. Chest wall kinematics can reliably estimate lung volumes^3^ but is impractical for routine use.

Dynamic chest radiography (DCR) is a low-dose, real-time digital imaging system that visualises the thorax over 10–20 s throughout the breathing cycle. A high-resolution flat panel detector (FPD) maximises image quality and field of view through digital reconstruction with minimal ionising radiation. DCR produces a ‘moving chest radiograph’, where thoracic structures can be observed throughout the breathing cycle. Using automatic detection of visible lung borders in the posteroanterior (PA) projection,^20^ the projected lung area (PLA) can be traced during different breathing phases. This technology has already been applied to diaphragm motion analysis in healthy volunteers,^21^ suspected diaphragm palsy,^22^ chronic obstructive pulmonary disease^23 24^ and individuals taking CF transmembrane conductance regulator (CFTR)-modifying drugs.^25^ PLA has been shown to correlate well with vital capacity in healthy volunteers^26^ and those with interstitial lung disease,^27^ but to our knowledge, this relationship has not been explored in pwCF.

DCR offers several advantages: first, unlike spirometry or plethysmography, it mirrors normal breathing, and since no mouthpiece or nose clip is required,^20^ it can be used in those with altered airway anatomy such as the presence of a tracheostomy. Second, image acquisition is rapid. Finally, DCR images are acquired at a distance from the performing radiographer, making it an appealing option in individuals with transmissible respiratory infections. The technology is Conformité Européenne marked for cineradiographic imaging in the UK and European Union and licensed for use in the USA.

The ability to combine chest imaging with rapid, non-aerosol-generating and physiological pulmonary function testing (PFT) may make DCR a useful adjunct to traditional measures of lung health in pwCF, a condition in which subtle markers of disease progression and treatment response are needed,^28^ especially in the era of CFTR modulator therapies.^29 30^ The aims of this study were to assess the feasibility of using PA and lateral DCR to calculate lung volume subdivisions in pwCF, explore the correlation between DCR lung areas and PFTs performed by WBP, and develop models to calculate lung volume subdivisions from DCR lung areas.

## Material and methods

### Study population

Between December 2019 and July 2020, consecutive adult (≥17-year-old) pwCF attending routine CF Annual Review at the Liverpool Heart and Chest Hospital Adult CF Unit were invited to participate in the study. Those undergoing pulmonary exacerbation of CF or other acute illnesses, those pregnant or lactating, those unable to perform reproducible spirometric manoeuvres to American Thoracic Society–European Respiratory Society (ATS-ERS) standards^31^ and those with significant radiation exposure in the past year (such as multiple CT scans) were excluded. Recruitment was censored at 20 participants (a recruitment flow chart can be found in online supplemental figure 1). Informed consent was obtained from all participants prior to participation in the study.

10.1136/bmjresp-2022-001309.supp1 Supplementary data

### Imaging protocol

Standing position PA and lateral DCR images were acquired using a dynamic radiography system (Konica Minolta, Tokyo, Japan), consisting of a CMP200DR 50 kW generator (CPI, Palo Alto, California, USA), AeroDR HD 17×17 FPD (Konica Minolta, Tokyo, Japan), Varian Rad-60 Saphire X-ray tube and Optica 60 collimator (Varian Medical Systems, Palo Alto, California, USA).

DCR exposure conditions for both PA and lateral images were as follows: source to image distance 200 cm, focal distance 180 cm, tube voltage 100 kV, tube current 80 mA, exposure duration of pulsed X-ray 4 ms, with an image capture rate of 15 fps. A tube filter of 1.0 mm Al, 0.1 mm Cu was used to filter out soft X-rays. Maximum achievable effective dose (ED) of ionising radiation for a combined PA and lateral image series, based on maximum DCR exposure settings, was calculated at 0.17 mSv; this compares with 0.014 mSv for a standard PA chest X-ray.

For PA DCR, after coaching to maximise concordance during image capture, images were acquired during the following sequential respiratory manoeuvres:

1. A tidal breath in and out.
2. A deep breath to full inspiration.
3. A passive breath out from full inspiration to end expiration.

For lateral views, 10 participants’ images were captured at full inspiration only, with the remainder repeating the PA image manoeuvres; all were captured in the left lateral position. All examinations were supervised by a respiratory physician (TSF, 3 years’ experience, or DN, 10 years’ experience).

### Image interpretation

**Figure 1:**
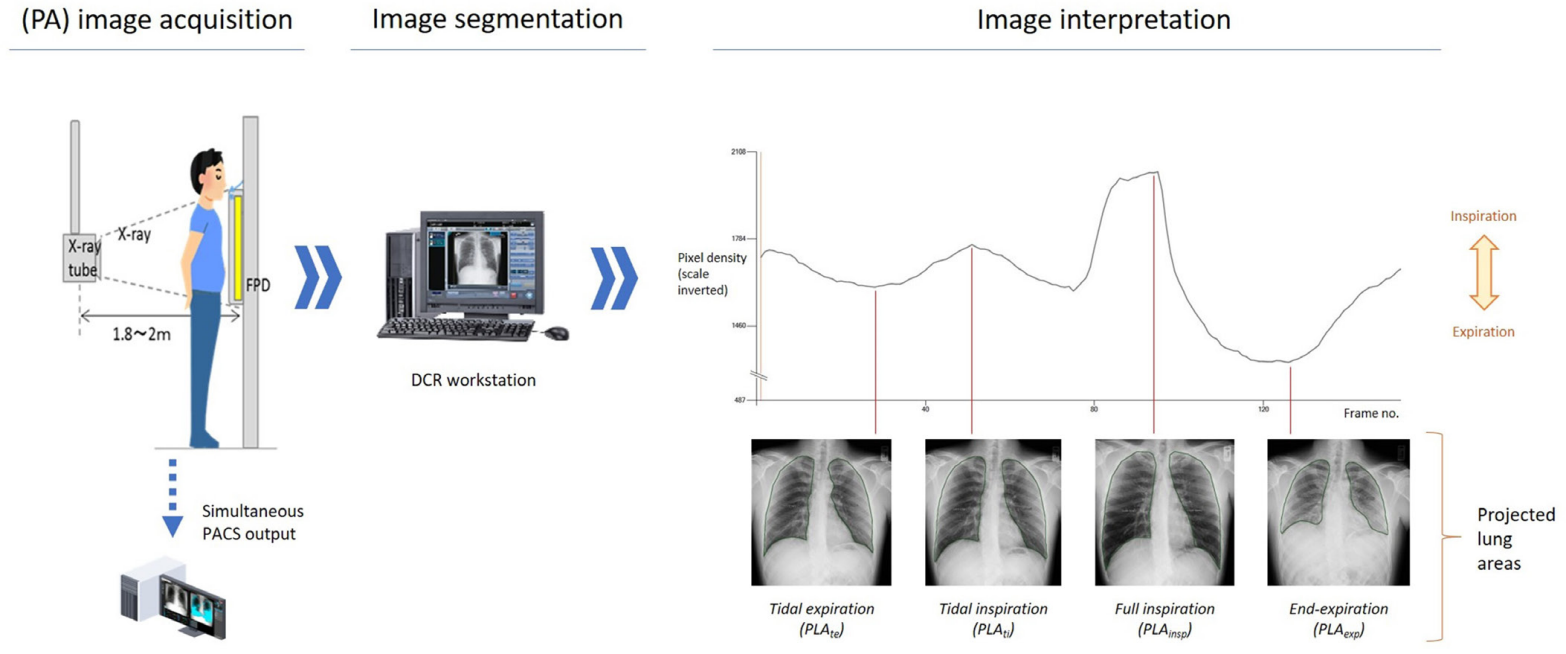
Dynamic chest radiography workflow and image segmentation. Respiratory phase is determined by the average pixel density during inspiration/expiration. DCR, dynamic chest radiography; FPD, flat panel detector; PA, posteroanterior; PACS, picture archiving and communication system; PLA, projected lung area.

**Figure 2:**
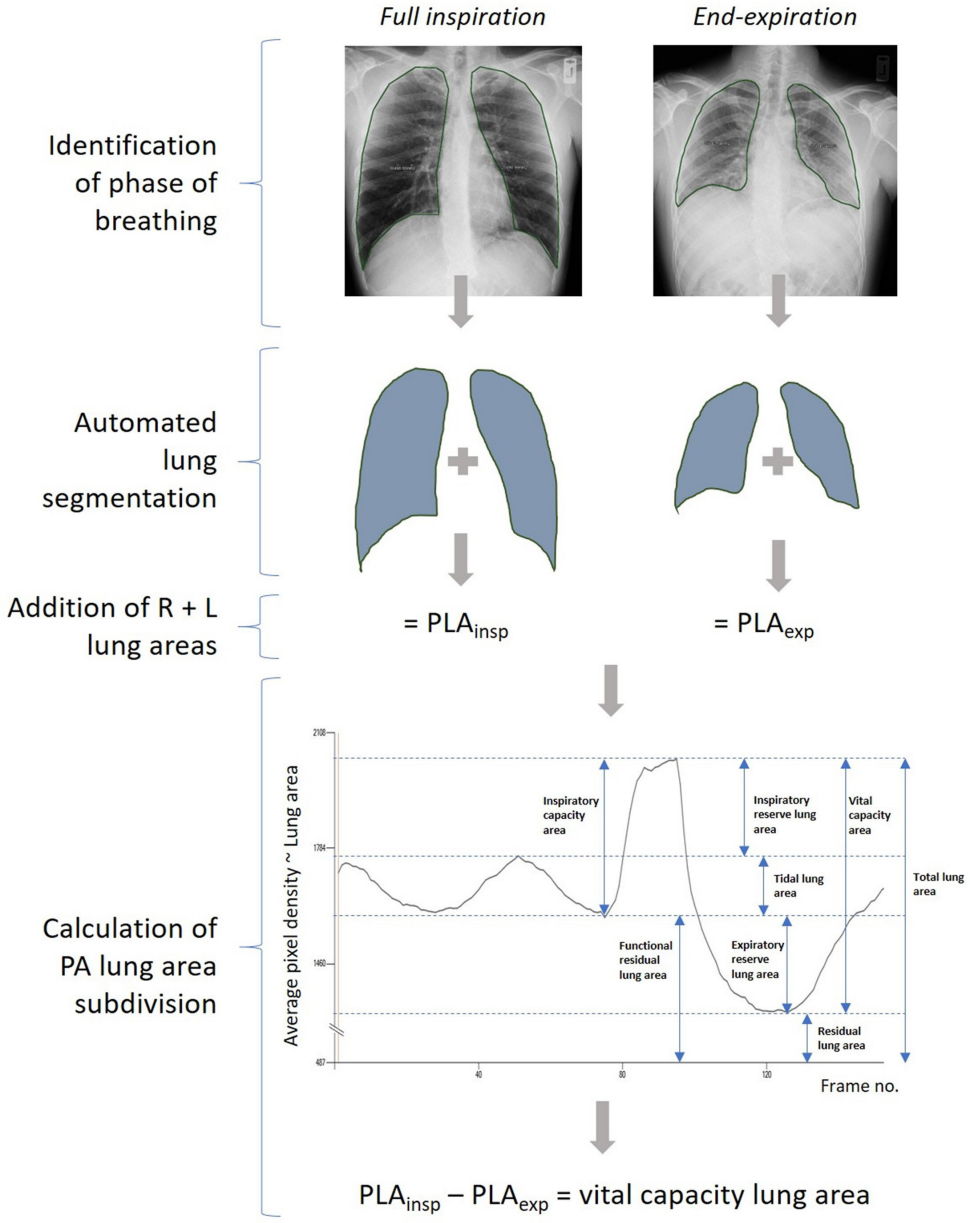
Example of dynamic chest radiography lung area subdivision calculation (vital capacity lung area). PA, posteroanterior; PLA, projected lung area.

DCR images were analysed using proprietary software (Konica Minolta). PLA was defined as the visible outline of lung on the image, excluding the area of lung concealed by the left heart border. An example of the automated lung tracing workflow is shown in figure 1, and a graphical example of a lung area subdivision calculation is shown in figure 2.

For PA images, PLAs were calculated automatically at maximum inspiration (PLA_insp_) and at the end of passive expiration (PLA_exp_). PLA_insp_ was determined by the software as the frame with the minimum average pixel density (that is, the ‘darkest’ image, at which the lungs are most expanded and thus least dense). PLA_exp_ was determined as the frame with the maximum average pixel density (that is, the ‘whitest’ image, at which the lungs are least expanded and thus most dense). Calculated lung borders on the PLA plots were checked for tracking errors by a respiratory physician and specialist cardiothoracic radiologist (TSF, CM) blinded to the lung function results, and corrected if any tracking errors were present. PLA was calculated at end-tidal inspiration (PLA_ti_) and expiration (PLA_te_) from lung borders traced manually by a respiratory physician (TSF) at these points (software to calculate PLA at any point during the breathing cycle is in development but was not available for use in this study). For lateral images, left and right PLAs were traced manually within the proprietary software by a respiratory physician (TSF), and the average value used.

### Pulmonary function testing

PFT was performed to ATS-ERS reproducibility criteria^31 32^ by an ARTP-registered pulmonary physiologist using a Lovemedical Bodystik body box (Lovemedical, Manchester, UK), on the same day as DCR images were acquired. Participants were requested not to use inhalers or airway clearance devices in between the two investigations. Global lung initiative reference values^33^ were used for plethysmography measurements. Forced expiratory volume in 1 s (FEV_1_), forced vital capacity (FVC), FEV_1_/FVC ratio, FRC (expressed as thoracic gas volume (TGV)), TLC, FVC, tidal volume (TV) and residual volume (RV) were recorded.

### Statistical analysis

Statistical analysis was carried out using Rstudio for R V.4.0.3 (R Foundation for Statistical Computing).

Using the sum of the left and right PLAs, PA PLA subdivisions were compared with their plethysmographic lung volume equivalent. For example, PLA_insp_ (that is, total lung area at full inspiration) was compared with TLC, and the difference between PLA_insp_ and PLA_te_ (inspiratory capacity area) was compared with IC (see online supplemental table 1).

10.1136/bmjresp-2022-001309.supp4 Supplementary data

Each defined PLA was compared with the corresponding lung volume subdivision using scatter plots and univariate analysis by Pearson’s product-moment correlation coefficient. The relationship between PLA and lung volume subdivision was explored using multiple linear regression, with height and weight (or body mass index (BMI)), age, and PA and lateral lung area subdivisions explored as predictor variables. Collinearity was tested for using variance inflation factors (see online supplemental table 2). Coefficients from significant models were applied to the PLA to calculate a predicted lung volume subdivision; due to the small number of participants (n=20), a maximum of two covariates was allowed per model. Residuals versus fitted value plots were used to visualise the created models.

TLC was also calculated using Pratt’s method,^10^ an established method to calculate TLC using maximal inspiratory plain PA and lateral chest X-rays, to validate the total lung area-derived TLC measurements calculated using the DCR images at full inspiration:

$$TLC=(0.67\times ((RightPLA+LeftPLA)\times (LateralPLA){)}^{3/4}))+320$$

Agreement between DCR and WBP-calculated TLC was assessed using the mean of the differences and Bland-Altman plots. To address issues of potential bias, mean difference distribution was assessed using distribution histograms; normality was assessed using the Kruskal-Wallis test and quantile–quantile plots.

Two-sided p values of less than 0.05 were considered significant. Descriptive, normally distributed statistics are reported where appropriate as mean±SD.

## Results

### Participant demographics and clinical characteristics

A total of 20 participants (age 30±9 years, per cent-predicted FEV_1_ 79±26, BMI 24±4 kg/m^2^, 6 female) were recruited. No adverse events were reported as part of the study, and no participants withdrew from the analysis. Participant characteristics are described in table 1. Results of DCR and pulmonary function studies are described in table 2.

**Table 1:** Clinical characteristics and anthropometric details

Characteristic	N (%)
Number of participants	20
Female	6 (30)
Male	14 (70)
Age (years), mean±SD	30±9
Body mass index (kg/m^2^), mean±SD	24±4
CF-related diabetes, n (%)	9 (45)
Chronic *Pseudomonas aeruginosa* colonisation	16 (80)
Smoker (current or ex), n (%)	0 (0)
CF genotype	
F508_del_ homozygotes F508_del_ heterozygotes Other	10 (50) 7 (35) 3 (15)

CF, cystic fibrosis.

**Table 2:** Results from pulmonary function tests and dynamic chest radiography

Variable	Value
Pulmonary function tests	
FEV_1_ (L)	3.2±1.1
FEV_1_ (% predicted)	79±26
FVC (L)	4.6±1.1
FVC (% predicted)	96±19
FEV_1_/FVC ratio	82±16
TLC (L)	6.6±1.3
IC (L)	2.9±0.8
RV (L)	1.9±0.7
TGV (L)	3.7±0.9
Dynamic chest radiography	
PLA_insp_ (cm^2^)	452±85
PLA_ti_ (cm^2^)	400±75
PLA_te_ (cm^2^)	351±73
PLA_exp_ (cm^2^)	319±63

Values expressed as mean±SD.

FEV_1_, forced expiratory volume in 1 s; FVC, forced vital capacity; IC, inspiratory capacity; PLA, projected lung area; RV, residual volume; TGV, thoracic gas volume; TLC, total lung capacity.

### DCR image acquisition

In two PA image series, tidal manoeuvres were judged by the clinicians (TSF, CM) to be suboptimal because a premature deep breath interrupted tidal expiration. In seven PA image series, passive expiration was judged by the clinician to have not been completed to a point in the breathing cycle consistent with residual volume by the time the mandated maximum time for X-ray exposure had been reached. Expiration to the point of maximum expiration normally takes 5–6 s but can take up to 15 s in individuals with obstructive spirometry,^31^ by which point the maximum allowable X-ray exposure time would already have been exceeded. The respective tidal and passive expiration phases of breathing for these individuals were excluded from the analysis, and the associated subdivisions calculated using the point of PLA_te_ were excluded (for example, RV). All participants completed all other manoeuvres successfully. All DCR images at the frame of PLA_insp_ were of sufficient quality to interpret as a standard PA chest radiograph so could be used for routine clinical diagnostic purposes.

### Correlations between DCR and plethysmography

**Figure 3:**
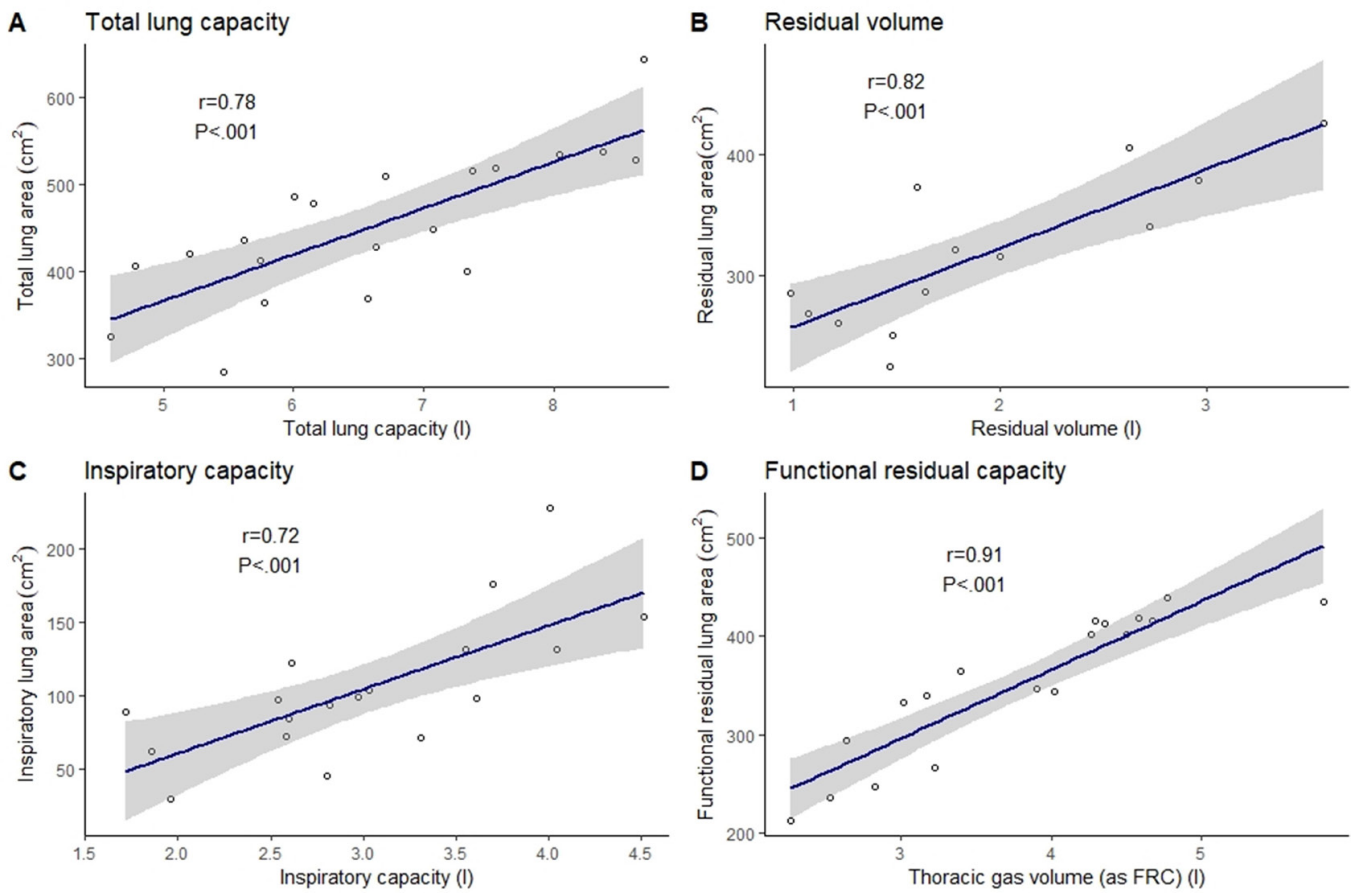
Correlation between projected lung areas and lung volume subdivisions calculated by whole-body plethysmography. A, total lung capacity with total lung area; B, residual volume with residual lung area; C, inspiratory capacity with inspiratory lung area; D, functional residual capacity (as thoracic gas volume) with functional residual lung area.

Total lung area correlated positively with TLC (r=0.78, p<0.001), functional residual lung area correlated positively with TGV (r=0.91, p<0.001), residual lung area correlated positively with RV (r=0.82, p<0.001) and inspiratory lung area correlated positively with IC (r=0.72, p<0.001) (see figure 3). FVC lung area and FVC showed a weak but significant correlation (r=0.67, p=0.01). No significant correlations were seen between tidal lung area and TV (r=−0.05, p=0.84), expiratory reserve lung area and expiratory reserve volume (r=0.49, p=0.13), or inspiratory reserve lung area and inspiratory reserve volume (r=0.47, p=0.05); therefore, these subdivisions were not included in the subsequent DCR lung volume modelling. Despite the strong and significant correlation between residual lung area and RV, due to the number of subjects (n=7) not reaching the point of the breathing cycle consistent with RV during DCR, modelling for RV was excluded from the analysis.

### Predictive models for lung volume subdivisions

TLC calculated by Pratt’s method was correlated positively with that calculated by WBP (r=0.87, p<0.001). Mean absolute error was 0.5 L, and root mean square error 0.66 L; a Bland-Altman plot of agreement is shown in online supplemental figure 2. Multiple linear regression modelling was performed with plethysmographic lung volume subdivisions as the predictor variable, and the corresponding lung area subdivisions (both PA and lateral), age, height and weight. Lateral images did not contribute significantly to any model, which may reflect their lower image detail (the same X-ray tube current and pulsed exposure duration as PA images were used despite greater tissue density, in order to limit ionising radiation exposure) and the superimposed structures such as the heart and hemidiaphragms present on a lateral radiograph (in the lateral projection, the hemidiaphragms and mediastinal structures overlap the visible lung areas), which may have interfered with accurate recording of lung area. Models are shown in table 3. As at most only 20 DCR series were acquired for each subdivision, model testing using a data subset or separate dataset was not possible. Residuals versus fitted value plots can be found in online supplemental figure 3.

10.1136/bmjresp-2022-001309.supp2 Supplementary data

10.1136/bmjresp-2022-001309.supp3 Supplementary data

**Table 3:** Linear regression models for lung volume subdivision calculation

Lung volume	n	Model	Multiple R^2^	Adjusted R^2^	P value	F
TLC	20	PLA_insp_ (cm^2^)×0.008+height (cm)×0.079–10.7	0.77	0.75	<0.001	30
IC	18	(PLA_insp_–PLA_te_) (cm^2^)×0.00011+height (cm)×0.030–2.27	0.56	0.50	0.002	10
FRC	18	(PLA_insp_–IC area) (cm^2^)×0.00011+height (cm)×0.012	0.83	0.81	<0.001	36

FRC, functional residual capacity; IC, inspiratory capacity; PLA, projected lung area; TLC, total lung capacity.

## Discussion

To our knowledge, this is the first study to explore the use of DCR in the assessment of lung volume subdivisions in adult pwCF, an important marker of lung health in this population. Despite the small sample size, correlations between DCR lung areas and plethysmographic lung volumes were significant and plausible. In particular, total lung area was correlated with TLC (r=0.78, p<0.001), functional residual lung area with TGV (r=0.91, p<0.001), residual lung area with RV (r=0.82, p<0.001) and inspiratory lung area with IC (r=0.72, p=0.001). We derived models that were able to calculate lung volume subdivisions in pwCF for TLC, RV and FRC, and demonstrated that lung areas calculated using DCR can be applied to Pratt’s method for calculation of TLC. These exploratory data suggest DCR may show promise as an adjunct to traditional methods of lung volume subdivision measurement.

The positive correlations observed between WBP-calculated and DCR-calculated TLC (r=0.87) were similar to those observed with ultra-low-dose CT in pwCF (TLC p=0.71) by Loeve *et al*^34^ and Lacerda *et al* (TLC r=0.71).^35^ Loeve *et al* found that ‘end-expiratory’ images acquired using CT were closer to FRC than RV, similar to our study in which full expiration to RV was not met in 7 out of 20 individuals performing DCR. The poor correlation seen between tidal lung area and plethysmographic TV may reflect the large influence that even a small increase in depth of breathing has on tidal lung area change. However, DCR can observe other measures of tidal breathing pattern such as diaphragm excursion and speed,^21^ and plethysmographic measurement of TV may be affected by the ‘observer effect’^36 37^ of respiratory pattern to a different degree than the less forced or repetitive technique of DCR. The effect of the mouthpiece and nose clip used in spirometry may also affect the depth, rate and pattern of tidal breathing^5–7^: DCR—in which no mouthpiece or other adjuncts are necessary—will be more representative of physiological breathing than a forced technique.

Although lateral DCR images were useful for calculating TLC by Pratt’s method, PA images alone correlated well with lung volumes, and during regression modelling, the lateral images were removed due to their low significance in predicting lung volume subdivisions. The increased radiation dose, acquisition time and increased difficulty in lung border tracing for lateral images suggest these may not be a clinically feasible part of DCR lung volume subdivision measurement. Future studies may wish to investigate the additional capabilities of DCR such as lung density assessment as a measure of air content, which might be incorporated into volume models in lieu of lateral imaging.^20^

In addition to lung volume calculation, CT markers of lung disease severity have been compared with lung function measurements in pwCF, with studies describing good correlations between FEV_1_ and airway thickening^38^ or Bhalla score.^39 40^ While similar structural comparisons were outside the remit of this work, DCR-calculated measures of disease severity—such as ventilation or perfusion^41^—may be an interesting focus to include in future work in pwCF.

This pilot study was limited primarily by its small size and lack of validation or control groups. Average BMI in this study was 24±4 kg/m^2^; at higher BMI, lung volumes may be negatively affected by obesity,^42^ but this relationship was not modelled for here. While the accuracy of the DCR volume measurement method falls below that of CT,^17^ the radiation dose is lower, suggesting DCR may be a more appropriate investigation for serial measurements. However, DCR will need to compete with recent advances in ultra-low-dose CT scanning techniques such as photon-counting CT^43^ and deep-learning reconstruction,^44^ which promise yet further ionising radiation dose reductions to a range comparable with that of a plain chest X-ray. Repetition of DCR after substandard breathing manoeuvres could not be performed in this study as it would have required further exposure to ionising radiation. This contrasts with plethysmography, which can be repeated, although taxingly for the patient. Concordance with DCR may improve with comprehensive coaching prior to imaging. Lower DCR frame rate might also allow longer imaging times and could potentially allow the use of DCR in children, in whom there is a need to develop markers of early CF lung disease.^45^ A 10-second DCR study at 6 fps gives an ED of 0.048 mSv.

## Conclusions

In conclusion, this pilot work demonstrates plausible associations between DCR lung areas and clinically important plethysmographic lung volume subdivisions. Larger studies to allow model validation, with a greater diversity in age, BMI and spectrum of CF lung disease, and the inclusion of healthy non-CF controls, will explore this technique further.

## Data Availability

Data are available upon reasonable request. Data generated as part of this work are available upon reasonable request from Dr Thomas FitzMaurice, Liverpool Heart and Chest Hospital NHS Foundation Trust.
