# Supplementary figures and images for: Feasibility of dynamic chest radiography to calculate lung volumes in adult people with cystic fibrosis: a pilot study
Source: BMJ Open Respir Res. 2023 May 5;10(1):e001309. doi: 10.1136/bmjresp-2022-001309 (PMC10163553; doi:10.1136/bmjresp-2022-001309)

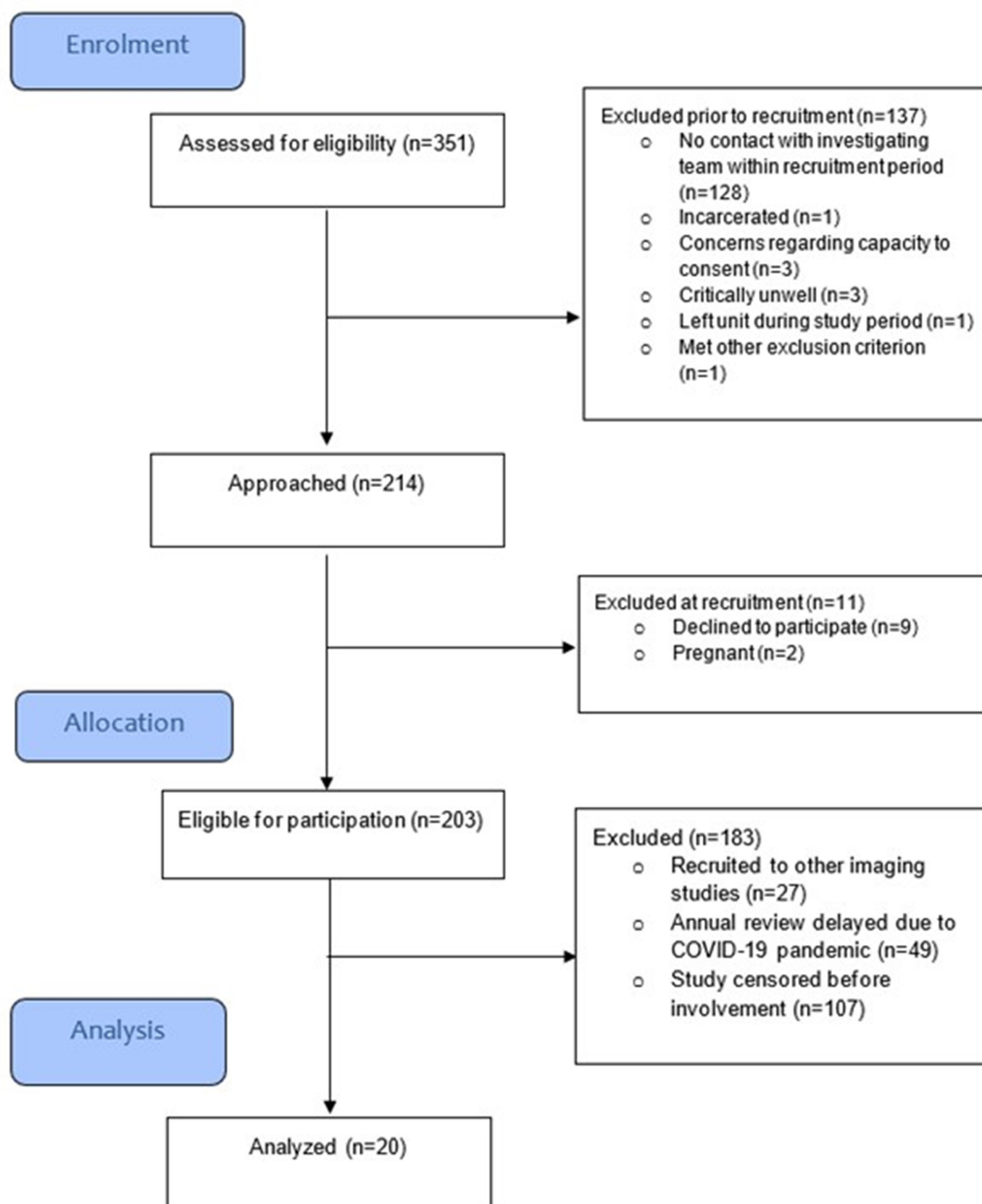

Supplement: Supplementary data [file bmjresp-2022-001309supp001.pdf]

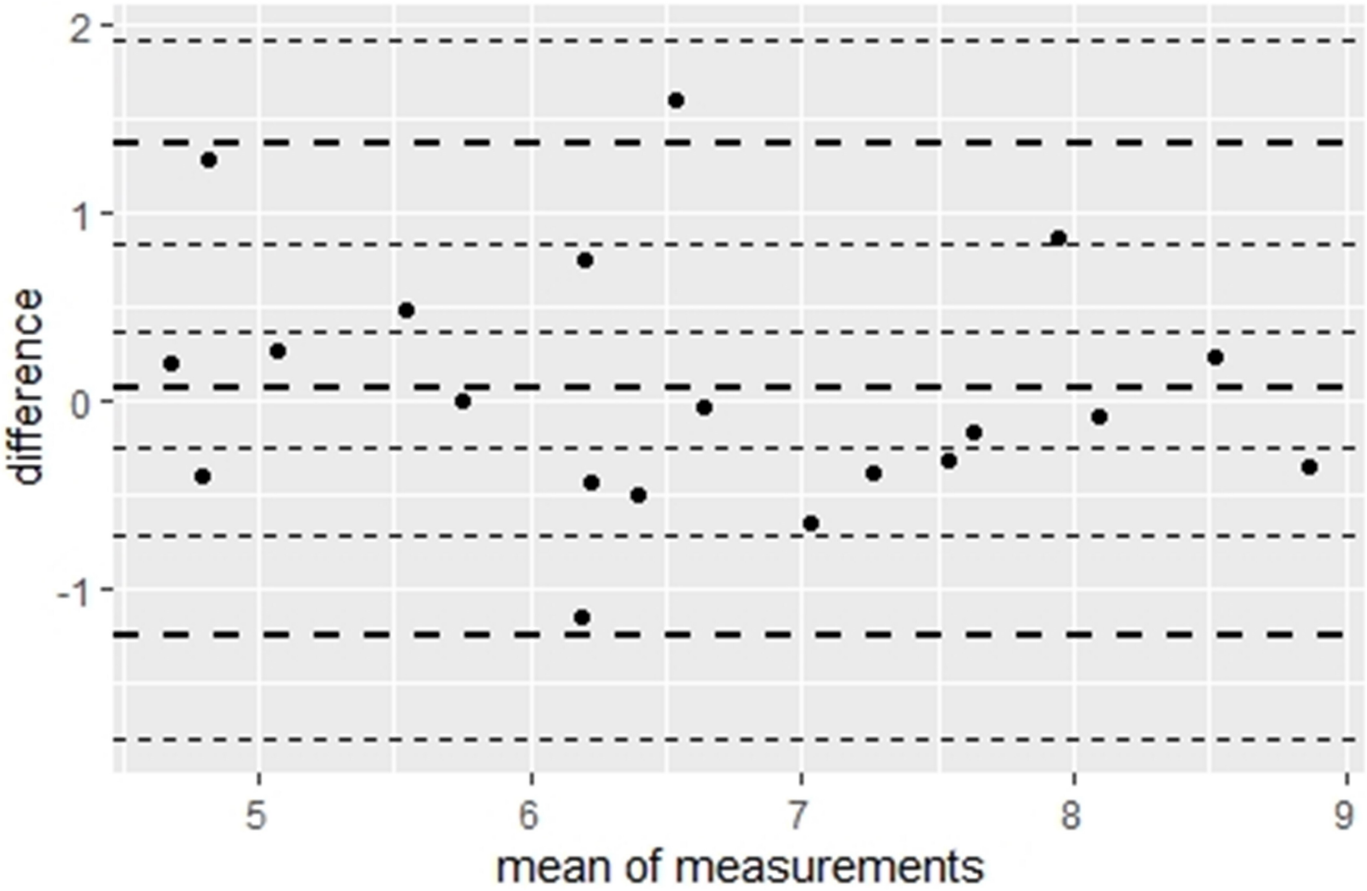

Supplement: Supplementary data [file bmjresp-2022-001309supp002.pdf]

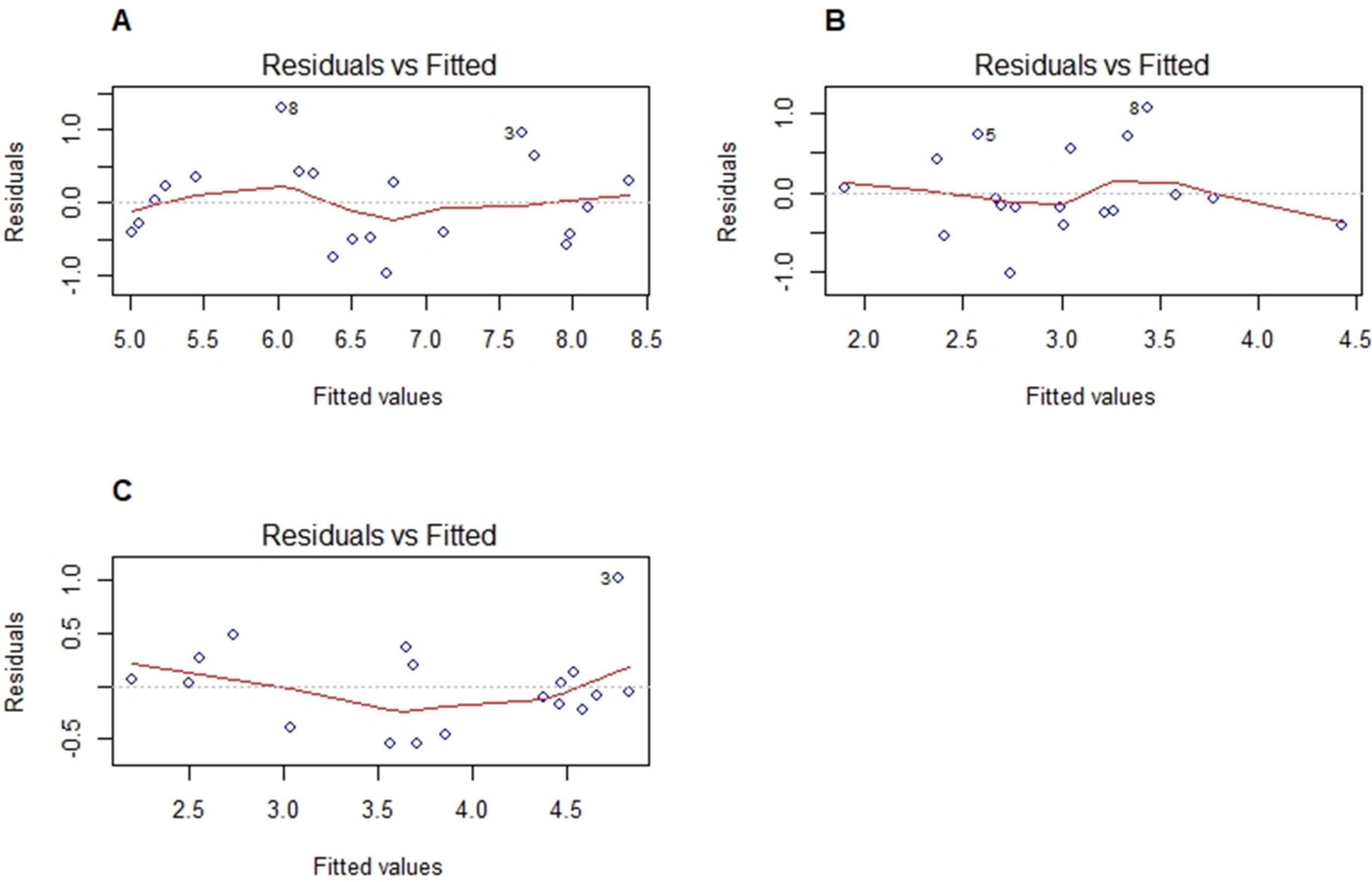

Supplement: Supplementary data [file bmjresp-2022-001309supp003.pdf]
