# Supplementary material for: Feasibility of dynamic chest radiography to calculate lung volumes in adult people with cystic fibrosis: a pilot study
Source: BMJ Open Respir Res. 2023 May 5;10(1):e001309. doi: 10.1136/bmjresp-2022-001309 (PMC10163553; doi:10.1136/bmjresp-2022-001309)
Supplement: Supplementary data [file bmjresp-2022-001309supp004.pdf]

Supplementary tables

Table S1 - Lung area subdivision calculations from lung area subdivisions

DCR lung area subdivision	Calculation	Plethysmographic volume equivalent
Total lung area	PLA_{insp}	TLC
Tidal lung area	PLA_{ti} - PLA_{te}	TV
Inspiratory capacity area	PLA_{insp} – PLA_{te}	IC
Functional residual capacity area	PLA_{insp} – IC area	TGV
Residual lung area	PLA_{exp}	RV

Table S2 - Variance inflation factors associated with each lung volume subdivision model

Volume subdivision	Variables	Variance inflation factor
TLC	PA total lung area<br>Height	1.328
IC	PA inspiratory capacity area<br>Height	1.035
FRC (TGV)	PA functional residual capacity area<br>Height	1.432
